# “I always get Panadol...there’s no hope here.” Healthcare services accessibility to incarcerated older adults.

**DOI:** 10.1093/geroni/igaf122.2664

**Published:** 2025-12-31

**Authors:** Akwasi Adjei Gyimah, Samuel Asante, Eric Frimpong, Mathias Adjei

**Affiliations:** Miami University Scripps Gerontology Center, Oxford, Ohio, United States; Northeastern State University, Tahlequah, Oklahoma, United States; University of Massachusetts Boston, Boston, Massachusetts, United States; Miami University, Oxford, Ohio, United States

## Abstract

The increasing number of Incarcerated older adults in Ghana is becoming a national issue for both policymakers and prison administrators. Currently, there are about 15,198 people incarcerated in various places in Ghana. These numbers result in having too many congestions and all these affect the quality of healthcare in prisons. The study sought to assess the quality of health care services for older adults in prisons in Ghana This study employed a qualitative approach involving an explorative descriptive design A focus group discussion was conducted among purposively selected older incarcerated adults. To be included, a participant must have been at least 50 years old and in prison for at least six months. A total of 28 older adults, comprising 18 males and 10 females were selected for participation in the study. The findings showed health care services accessibility in prison facilities remains a significant issue. Participants acknowledged that healthcare workers in prisons have limited knowledge and practice skills to effectively address their healthcare needs. They also pointed to the lack of medical equipment in prisons and stigma as additional factors that adversely affect their healthcare experiences in prisons. Participants generally noted that the quality of health services in prisons was substandard compared to that provided to the general public. The study adds to the literature on older incarcerated adults in Africa and how their healthcare accessibility can be improved. The study underscores the need for policy changes and practices that could enhance the healthcare experiences of older adults in prisons.

